# Ultra-Wideband Vertical Transition in Coplanar Stripline for Ultra-High-Speed Digital Interfaces

**DOI:** 10.3390/s24103233

**Published:** 2024-05-19

**Authors:** Mun-Ju Kim, Jung-Seok Lee, Byung-Cheol Min, Jeong-Sik Choi, Sachin Kumar, Hyun-Chul Choi, Kang-Wook Kim

**Affiliations:** 1 School of Electronic and Electrical Engineering, Kyungpook National University, Daegu 41566, Republic of Korea; dranswn@knu.ac.kr (M.-J.K.); j.seok1020@knu.ac.kr (J.-S.L.); minbc4658@knu.ac.kr (B.-C.M.); jeongsik2@knu.ac.kr (J.-S.C.); hcchoi@ee.knu.ac.kr (H.-C.C.); 2 Department of Electronics and Communication Engineering, Galgotias College of Engineering and Technology, Greater Noida 201310, India; gupta.sachin0708@gmail.com

**Keywords:** vertical transition, ultra-high-speed interface, conformal mapping, coplanar stripline, ultra-wideband

## Abstract

A design method for an ultra-wideband coplanar-stripline-based vertical transition that can be used for ultra-high-speed digital interfaces is proposed. A conventional via structure, based on a differential line (DL), inherently possesses performance limitations (<10 GHz) due to difficulties in maintaining constant line impedance and smooth electric field transformation, in addition to the effects of signal skews, FR4 fiber weave, and unbalanced EM interferences. DL-based digital interfaces may not meet the demands of ultra-high-speed digital data transmission required for the upcoming 6G communications. The use of a coplanar stripline (CPS), a type of planar balanced line (BL), for the vertical transition, along with the ultra-wideband DL-to-CPS transition, mostly removes the inherent and unfavorable issues of the DL and enables ultra-high-speed digital data transmission. The design process of the transition is simplified using the analytical design formulas, derived using the conformal mapping method, of the transition. The characteristic line impedances of the transition are calculated and found to be in close agreement with the results obtained from EM simulations. Utilizing these results, the CPS-based vertical transition, maintaining the characteristic line impedance of 100 Ω, is designed and fabricated. The measured results confirm its ultra-wideband characteristics, with a maximum of 1.6 dB insertion loss and more than 10 dB return loss in the frequency range of DC to 30 GHz. Therefore, the proposed CPS-based vertical transition offers a significantly wider frequency bandwidth, i.e., more than three times that of conventional DL-based via structures.

## 1. Introduction

With the advent of the fourth industrial revolution, technologies for 5G communications are rapidly under development, and a variety of research on improving high-capacity and high-speed digital data transmissions is under way to prepare for the upcoming 6G communications. Technologies for 6G communications such as the Internet of Things (IoT), autonomous driving, and artificial intelligence (AI) demand digital data transmission speeds ranging from hundreds of Gbps to several Tbps [1,2].

Digital signals are in the form of rectangular waves and occupy a significant frequency bandwidth, comprising a main signal frequency and its multiple harmonic components. The conventional transmission line used for high-speed digital transmission is a differential line (DL), consisting of two closely spaced microstrip lines placed parallel to each other with two signal lines and a ground plane. The two lines of the DL carry signals with opposite polarities, forming a differential signal, and the typical characteristic line impedance used for the DL is 100 Ω. The differential signal allows it to operate at lower voltage levels and typically exhibits relatively high immunity to external noises, making it widely used in high-speed digital transmission lines [3]. However, as the transmission speed of the digital signal increases, significant issues may arise due to the structural and electrical limitations of the DL. When there is a length mismatch between the two signal lines or when there is unequal electromagnetic interference, a signal skew occurs, resulting in electromagnetic interference, signal integrity degradation, and malfunctions in receiving devices. Additionally, for high-speed digital transmission, it is necessary to minimize signal reflections by maintaining a constant impedance along the transmission line.

In the complex structures of a digital circuit board, high-speed digital lines based on the DL often have discontinuities, such as variations in the line width, the presence of ground apertures, and sections using vias, therefore causing signal skews and reflections. Specifically, using vias in high-speed digital circuits based on DL presents difficulties in controlling impedance discontinuity. The vias are often used in multilayer substrates for interlayer transitions in circuits and are employed for pad layouts in high-density PCBs where numerous surface-mount devices are connected in limited spaces [4]. Additionally, the vias are utilized in the connection of various devices to circuit boards in interface designs [5].

In these cases, electric field distributions due to vias can be abnormal, and the stray electric field lines, generated as the vias pass through the ground plane, can cause inter-layer radiation, affecting adjacent lines [6]. For instance, a commercially available USB 3.0 interface based on the DL, which operates up to 10 GHz per line, shows limitations at higher data transmission speeds [7]. Previously, complex digital signal processing techniques were employed to enhance digital data transmission speeds [8]. However, these approaches had the drawback of causing increased overall power consumption within a system.

Various studies to improve digital transmission speed have been conducted by optimizing via parameters such as the via diameter, via gap, via pad diameter, and ground aperture diameter to control the impedance discontinuity in the via structures [9,10], and by minimizing signal reflection and radiation through adding ground vias or adopting various ground via patterns [11,12,13]. Research has also focused on a new DL-based via structure with a flat-faced design as an alternative to optimizing the cylindrical via structures in DLs [14]. Additionally, studies have focused on analyzing the electromagnetic coupling between signal vias and ground planes with a proposal of analytical models for the via structures [15,16]. Despite these research efforts to minimize reflection and maintain signal integrity in the via structures, whenever the structure of the digital circuit changes, complex via structures with numerous parameters should be redesigned and re-optimized using a sophisticated design process. The performance of a single DL-based via structure could be enhanced through optimization of the ground structure with more ground vias (e.g., 2~6) [11,13], but this required significantly more space on the circuit boards. In addition, this configuration may significantly complicate the design process for multiple closely spaced vertical transitions to achieve similar high performances within the limited space of digital circuit boards. To achieve ultra-high-speed digital transmission for high-capacity data, it is necessary to have an ultra-wideband vertical transition with a simple structure that maintains constant impedance and minimizes radiation and interference with adjacent lines in the via structures.

On the other hand, in order to significantly enhance the digital transmission speed, ultra-wideband transmission lines and structures were proposed, enabling the sustenance of signal integrity in high-speed digital data transmission without incurring additional power consumption, thus making it a favorable solution [17]. For ultra-high-speed digital data transmission on typical PCBs, ultra-wideband transitions from a DL to a balanced line (BL), such as a coplanar stripline (CPS) and parallel stripline (PSL), were proposed. These BL-based transmission lines, which support differential signaling, provide effective noise rejection, common-mode signal elimination, and self-phase recovery properties [17]. Here, a CPS is a transmission line with only two signal lines placed on the same plane and is often utilized as a balanced antenna balun [18]. Each signal line acts as a ground reference for the other, resulting in strong electromagnetic coupling between the two lines.

**Figure 1:**
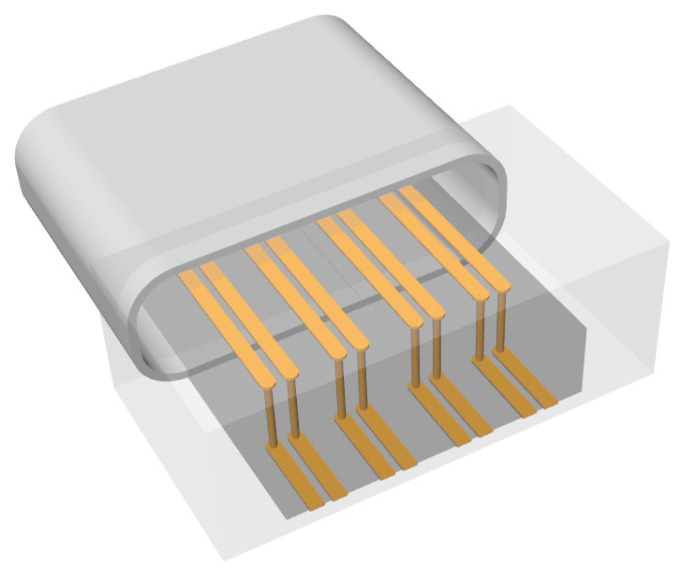
Perspective view of an ultra-high-speed digital interface using the proposed CPS-based vertical transitions.

In this paper, an ultra-wideband CPS-based vertical transition for ultra-high-speed digital interfaces is proposed. The proposed vertical transition based on CPS has simple via structures, and analytical design formulas based on conformal mapping are used for rapid and straightforward line impedance calculations and adjustments. The proposed interface structure provides constant line impedance and smoothly transforms electric field distributions, thereby achieving the ultra-wideband characteristics suitable for an ultra-high-speed digital interface, as illustrated in Figure 1. Also, the configuration and performance of the proposed vertical transition are compared with those of previously reported via structures in Table 1.

## 2. Design of a Conventional DL-Based via Structure

**Figure 2:**
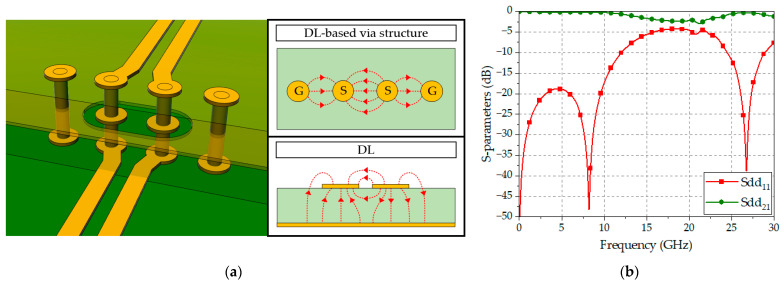
A conventional DL-based via structure: (**a**) Perspective view and simplified electric field distributions; (**b**) EM-simulated S-parameters.

An example of a conventional DL-based via structure is shown in Figure 2a, where two signal lines are vertically connected to upper signal lines using the corresponding signal vias and two ground vias, symmetrically placed at both sides of the signal vias, forming a total of four vias [7]. A ground aperture is also formed, encircling the two signal vias on the middle plane. In this case, spurious electromagnetic coupling occurs between the signal vias and the ground plane, leading to unwanted impedance variations and interlayer radiation. Moreover, the two ground vias need to be maintained at an appropriate distance from the signal vias to ensure the constant line impedance of the via structure. Consequently, controlling impedance discontinuity in the DL-based via structure imposes difficulties due to its complex design.

Furthermore, while the electric field distributions between the two signal lines and the ground plane in DLs are mostly vertical, those between the signal vias and the ground vias in the DL-based via structure are mainly horizontal. This results in an abrupt change in the electric field distributions when the DL and the DL-based via structure are connected. Figure 2b shows a result of a 3D EM simulation for the DL-based via structure in a multilayer substrate, showing a return loss of more than 10 dB in the frequency range of DC to 12 GHz, while the performance degrades rapidly after 12 GHz.

## 3. Design of a CPS-Based Vertical Transition

**Figure 3:**
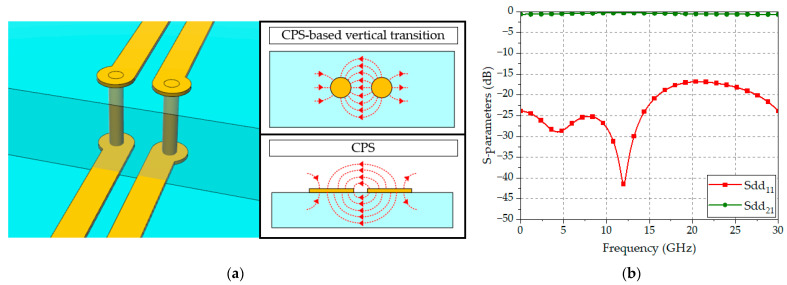
A proposed CPS-based vertical transition: (**a**) Perspective view and electric field distributions; (**b**) EM-simulated S-parameters.

A perspective view of a CPS-based vertical transition and its simplified electric field distributions are shown in Figure 3a. Use of the CPS as the digital transmission line simplifies the configuration of the vertical transition, i.e., only two signal vias are connected to the corresponding signal lines, as shown in Figure 3a, without requiring extra ground planes or vias. The line impedance of the via pair can be easily adjusted by modifying the radius and the gap distance between the vias. Additionally, the electric field lines of the CPS are concentrated between the two signal lines, smoothly transforming to those of the CPS-based vertical transition. Consequently, by using the proposed transition, it is possible to maintain the appropriate line impedance through simple adjustments along with smooth electric field transformation, thus providing the ultra-wideband characteristics suitable for high-capacity data transmission in an ultra-high-speed digital interface. Figure 3b depicts the result of the EM simulations of the proposed CPS-based vertical transition in a multilayer substrate, showing a return loss of more than 16.8 dB and an insertion loss of less than 0.6 dB in the frequency range of DC-30 GHz, demonstrating performance suitable for the implementation of ultra-high-speed digital interfaces.

**Figure 4:**
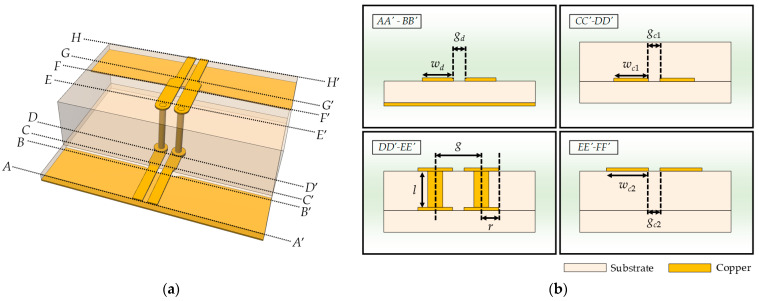
(**a**) Perspective view; (**b**) Cross-sectional views of the proposed CPS-based vertical transition.

A detailed configuration of the proposed CPS-based vertical transition with design parameters is illustrated in Figure 4a,b. The proposed vertical transition consists of seven distinct sections. In the *AA′*-*BB′* section, the bottom DL lines of the transition are supposed to contact the DL signal traces on the motherboard of a PCB, with a line width of *w_d_* and a gap of *g_d_*. The *BB′*-*CC′* section is a transition, where the line structure changes from a DL to a CPS. The width of the signal lines increases linearly with a constant gap width, and the ground aperture width also increases. In the *CC′*-*DD′* section, two signal lines with a width of *w_c_*_1_ and a gap of *g_c_*_1_ form a conventional CPS. In the *DD′*-*EE′* section, two cylindrical vias with a radius of *r* are placed with a gap distance of *g*. The length of the vias, denoted as *l*, is adjusted proportionally to the height of the interface substrate. The *EE′*-*FF′* section represents a CPS section on the upper part of the interface with a width of *w_c_*_2_ and a gap of *g_c_*_2_. In the *FF*-*GG′* section, the CPS transforms back to the DL, maintaining the constant gap while reducing the line width. The ground aperture width decreases until reaching the *GG′*-*HH′* section, where it finally transits back to the conventional DL with a line width of *w_d_* and a gap of *g_d_*.

### 3.1. Electric Field Distributions

**Figure 5:**
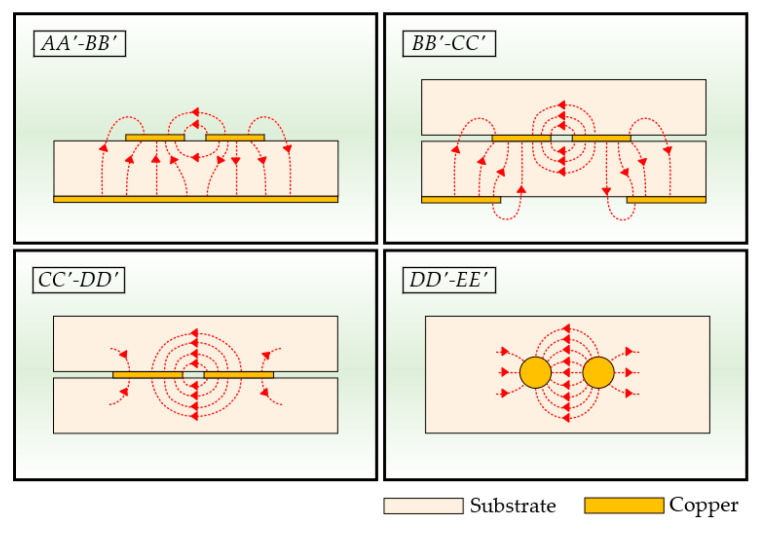
Electric field distributions at the representative cross-sectional stages of the proposed CPS-based vertical transition.

Figure 5 shows simplified electric field distributions at representative sections of the proposed CPS-based vertical transition. Due to the symmetry of the vertical transition, the electric field distributions of the *AA′*-*EE′* region are shown. In the *AA′*-*BB′* section, the electric field distribution is similar to that of the conventional DL, where the electric field lines are mainly concentrated between each signal line and the ground plane and partially distributed between the two signal lines of opposite polarities. As the structure transforms from DL to CPS in the *BB′*-*CC′* section, the ground aperture widens. Thus, fringing fields pass through the ground aperture, resulting in an increased intensity of the electric field lines between the signal lines. The *CC′*-*DD′* section shows the conventional CPS electric field distribution, where the electric field lines are concentrated between the two signal lines. In the *DD′*-*EE′* section, where the vias are placed, the cross-section of the two signal vias can be represented by two circular conductors. In a similar way to the conventional CPS, the two signal vias act as the ground reference for each other, and the electric field lines are mainly distributed between two signal lines.

### 3.2. Cross-Sectional Models and Analytical Formulas

**Figure 6:**
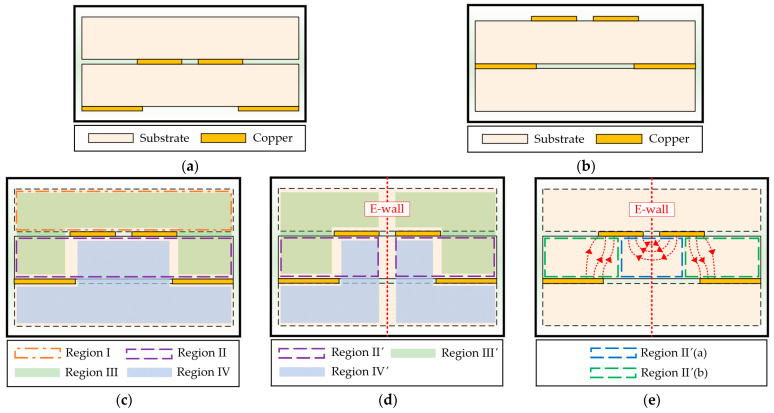
Cross-sections of the DL-to-CPS transition for analytical modeling: (**a**) Cross-section of the *BB′-CC′* section; (**b**) Cross-section of the *FF′-GG′* section; (**c**) Four analysis regions; (**d**) Analysis regions divided by an E-wall; (**e**) Region II’(a) and Region II’(b).

The *BB′*-*CC′* and *FF′*-*GG′* sections of the proposed CPS-based vertical transition in Figure 4 represent the DL-to-CPS transition. The analytical design formulas for the characteristic line impedance of this transition were described in [17], but the dielectric regions are slightly modified in this paper. To derive the analytical formula for the capacitance of each cross-section of the transition using the conformal mapping method, it is assumed that quasi-TEM or TEM electromagnetic fields are formed at each cross-section of the transition. The cross-sections of the transition and four analysis regions used for the conformal mapping are illustrated in Figure 6. The analytical formulas for the capacitances are presented in Table 2.

Region I represents a region above the two signal lines in the transition. In the *BB′*-*CC′* section, a dielectric substrate is placed for the CPS-based vertical transition, and the relative permittivity $\varepsilon_{r}$ should also be considered (Figure 6a,c), but in the *FF′*-*GG′* section, the air region is present above the two signal lines, and only the air permittivity $\varepsilon_{0}$ is considered (Figure 6b,c). The capacitance $C_{1}$ in the *BB′-CC′* section is equivalent to the inner region of CPS, and in the *FF′-GG′* section, the capacitance $C_{1}^{\prime }$ is equivalent to the upper region of CPS, where K is the elliptic integral of the first kind. The modulus $k_{1}$ and complementary modulus $k_{1}^{\prime }$ are the same as the moduli for Type 2 in [19]. Region II represents a region inside the dielectric medium. Since the structure is symmetric with respect to an E-wall, only half of the region (Region II’) is analyzed, as shown in Figure 6d. Region II’ is further divided into Region II’(a) for the capacitance $C_{2a}$ between the signal line and the E-wall and Region II’(b) for the capacitance $C_{2b}$ between the signal line and the ground plane, as shown in Figure 6e. The modulus $k_{2a}$ and complementary modulus $k_{2a}^{\prime }$ are equivalent to the moduli for Type 2 in [19], and the modulus $k_{2b}$ and complementary modulus $k_{2b}^{\prime }$ are equivalent to the moduli for Type 3 in [19]. Region III represents a region of fringing fields between the two signal lines and the ground plane, while Region IV represents a region of fringing fields passing through the ground aperture. The electric field lines in Region III of the *BB′-CC′* section and Region IV of the *FF′-GG′* section are primarily distributed in the dielectric medium. On the other hand, in Region IV of the *BB′-CC′* section and Region III of the *FF′-GG′* section, the electric field lines are primarily distributed in the air. Due to the symmetry with respect to the E-wall, half of the regions (Region III’ and Region IV’) can be used to calculate the capacitances $C_{3}$, $C_{4}$ for the *BB′-CC′* section and $C_{3}^{\prime }$, $C_{4}^{\prime }$ for the *FF′-GG′* section, respectively. The moduli $k_{3}$, $k_{4}$ and complementary moduli $k_{3}^{\prime }$, $k_{4}^{\prime }$ are the same as the moduli for Type 5 in [19].

Through these calculations, the effective permittivity of the *BB′*-*CC′* section is determined using (1), and the effective permittivity of the *FF′*-*GG′* section is obtained using (2). Correspondingly, the characteristic line impedances for these sections can be obtained using (3) and (4), respectively.

(1) $$\varepsilon_{eff}=\frac{C_{1}{/2+C}_{2a}{+C}_{2b}{+C}_{3}{+C}_{4}}{C_{1}{/2\varepsilon }_{r}{+C}_{2a}{/\varepsilon }_{r}{+C}_{2b}{/\varepsilon }_{r}{+C}_{3}{/\varepsilon }_{r}{+C}_{4}}$$

(2) $$\varepsilon_{eff}^{\prime }=\frac{C_{1}^{\prime }{/2+C}_{2a}{+C}_{2b}{+C}_{3}^{\prime }{+C}_{4}^{\prime }}{C_{1}^{\prime }{/2+C}_{2a}{/\varepsilon }_{r}{+C}_{2b}{/\varepsilon }_{r}{+C}_{3}^{\prime }{+C}_{4}^{\prime }{/\varepsilon }_{r}}$$

(3) $$Z_{0}=2\frac{{120\;\pi \;\varepsilon }_{0}}{\sqrt{\varepsilon_{eff}}\left(C_{1}{/2\varepsilon }_{r}{+C}_{2a}{/\varepsilon }_{r}{+C}_{2b}{/\varepsilon }_{r}{+C}_{3}{/\varepsilon }_{r}{+C}_{4}\right)}$$

(4) $$Z_{0}^{\prime }=2\frac{120\;\pi \;\varepsilon_{0}}{\sqrt{\varepsilon_{eff}^{\prime }}\left(C_{1}^{\prime }{/2+C}_{2a}{/\varepsilon }_{r}{+C}_{2b}{/\varepsilon }_{r}{+C}_{3}^{\prime }{+C}_{4}^{\prime }{/\varepsilon }_{r}\right)}$$

**Figure 7:**
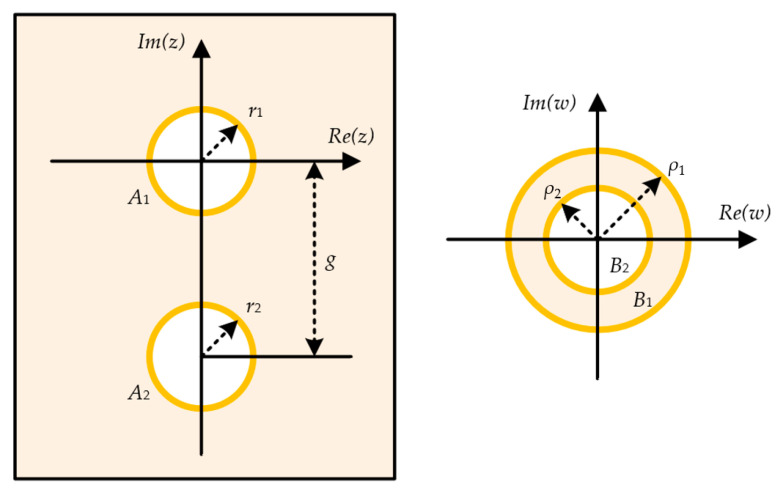
Cross-section of the vertical via transition for analytical modelling.

The *DD′*-*EE′* section represents a region with vertical vias, where the cross-section of the two vias connecting to the CPS lines can be represented by two circular conductors. The cross-sectional configuration of the vias for applying the conformal mapping method is depicted in Figure 7 [20]. The two circles, *A*_1_ and *A*_2_, are placed on the *z*-plane with radii *r*_1_ and *r*_2_, respectively, and their centers are separated by a gap distance *g*. To calculate the capacitance between the two conductors, the two circles *A*_1_ and *A*_2_ on the *z*-plane can be transformed into two concentric circles *B*_1_ and *B*_2_ on the *w*-plane through conformal mapping.

The radii *ρ*_1_ and *ρ*_2_ of the two concentric circles on the *w*-plane can be obtained using (6) and (7), respectively. The configuration composed of the two concentric circles is equivalent to the cross-section of a coaxial cable, enabling calculation of the capacitance C using (8). Using the calculated capacitance C, we can derive the characteristic line impedance $Z_{0}$ of the via section of the CPS-based vertical transition using (9). In this proposed transition, the signal vias consist of two cylindrical conductors with an equal radius (*r*_1_ = *r*_2_).

(5) $$N_{\;}=\frac{\sqrt{{{(r}_{1}^{2}{+r}_{2}^{2}-g^{2})}^{2}{-\;4\;r}_{1}^{2}{\;r}_{2}^{2}}}{2\;g}$$

(6) $$\rho_{1}{=Ne}^{{\sin h}^{-1}{(N/r}_{1})}$$

(7) $$\rho_{2}{=Ne}^{{-\sin h}^{-1}{(N/r}_{2})}$$

(8) $$C=\frac{{2\;\pi \;\varepsilon }_{r}}{{\ln(\rho }_{1}{/\rho }_{2})}$$

(9) $$Z_{0}=\frac{120}{C}\sqrt{\varepsilon_{r}}$$

### 3.3. Characteristic Line Impedance

The proposed CPS-based vertical transition, as shown in Figure 4a, consists of two similar DL-to-CPS transition sections (*BB′*-*CC′* and *FF′*-*GG′*) and a vertical via transition section (*DD′*-*EE′*). To verify the accuracy of the characteristic line impedance formulas derived using the conformal mapping method for the CPS-based vertical transition, the calculated line impedances are compared with the EM-simulated results. For the EM simulations, a commercial 3D EM simulator (CST Microwave Studio) is used. The proposed transition is designed using the 8 mil and 32 mil Rogers 4003C substrates ($\varepsilon_{r}=3.38$, tanδ=0.0027).

**Figure 8:**
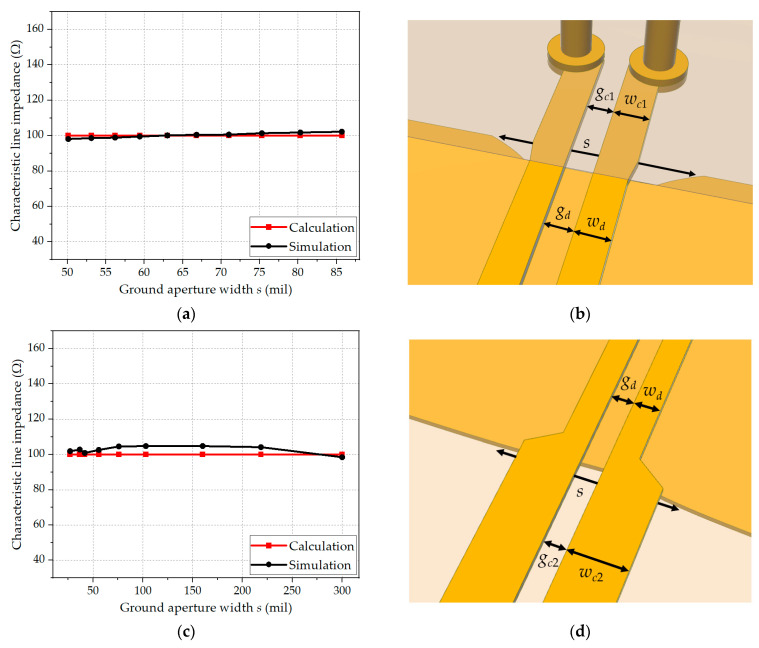
Calculated and EM-simulated characteristic line impedances of the DL-to-CPS transition: (**a**) Characteristic line impedance of the *BB′-CC′* section; (**b**) Design parameters of the *BB′-CC′* section (*w_d_* = 11.4 mil, *w_c_*_1_ = 13 mil, *g_d_* = *g_c_*_1_ = 5 mil); (**c**) Characteristic line impedance of the *FF′-GG′* section; (**d**) Design parameters of the *FF′-GG′* section (*w_d_* = 11.4 mil, *w_c_*_2_ = 24.5 mil, *g_d_* = *g_c_*_2_ = 5 mil).

First, the accuracy of analytical design Formulas (3) and (4) for the DL-to-CPS transition sections (*BB′*-*CC′* and *FF′*-*GG′*) is verified by comparing them with the EM-simulated results. The dimensions of the DL-to-CPS transitions can be flexibly adjusted to fit typical DL-based PCBs. Using the analytical formulas given in (3) and (4), the desired line gaps, line widths, and aperture widths of the DL and CPS lines can be easily determined to form a constant line impedance of 100 Ω. Figure 8a shows the characteristic line impedance of the *BB′-CC′* section as a function of the ground aperture width *s* with the 8 mil Rogers 4003C substrate. The design parameters of the section are shown in Figure 8b. The errors between the calculated characteristic line impedances and the EM simulation results are within 2.2%. The characteristic line impedance of the *FF′-GG′* section as a function of the ground aperture width *s* with the 8 mil Rogers 4003C substrate is shown in Figure 8c, and Figure 8d shows the design parameters of the section. The errors between the calculated characteristic line impedances and the EM simulation results are within 4.8%. These deviations in each section are possibly caused by an overlap area between Regions II and IV.

**Figure 9:**
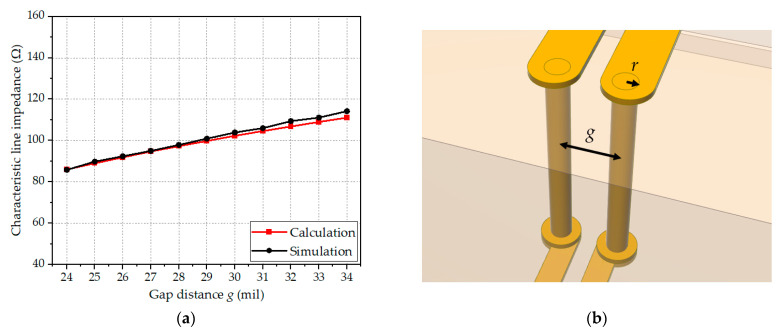
Calculated and EM-simulated characteristic line impedances of the vertical via transition: (**a**) Characteristic line impedance of the *DD′-EE′* section; (**b**) Design parameters of the *DD′-EE′* section (*r*_1_ = *r*_2_ = 6 mil).

Next, for the vertical via transition section (*DD′*-*EE′*), which is composed of the two cylindrical conductors with a gap distance *g*, the calculated characteristic line impedances using the analytic formula in (9) are compared with the EM-simulated results. Figure 9a shows the characteristic line impedance as a function of the gap distance *g* between the via centers, and Figure 9b shows the design parameters of the section. The via radius (*r*_1_ = *r*_2_) is set to 6 mil by considering manufacturing tolerances. The height of the vias is 72 mil using multiple Rogers 4003C substrates (one 8 mil and two 32 mil substrates). The errors between the calculated line impedances and the simulation results are within 3.0%. As the gap between the vias is increased, the error in the characteristic line impedance is also slightly increased, which could be attributed to the difference in calculation boundary between the analytical model and the EM simulation.

Therefore, since the accuracy of the analytical design formulas given in (3), (4), and (9) is verified as shown in Figure 8 and Figure 9, for the design of the proposed CPS-based vertical transition, the values of widths, gaps, and via radius are selected to maintain the characteristic line impedance of 100 Ω in all sections. Table 3 lists the design parameters of the proposed transition.

## 4. Fabrication and Measurements

**Figure 10:**
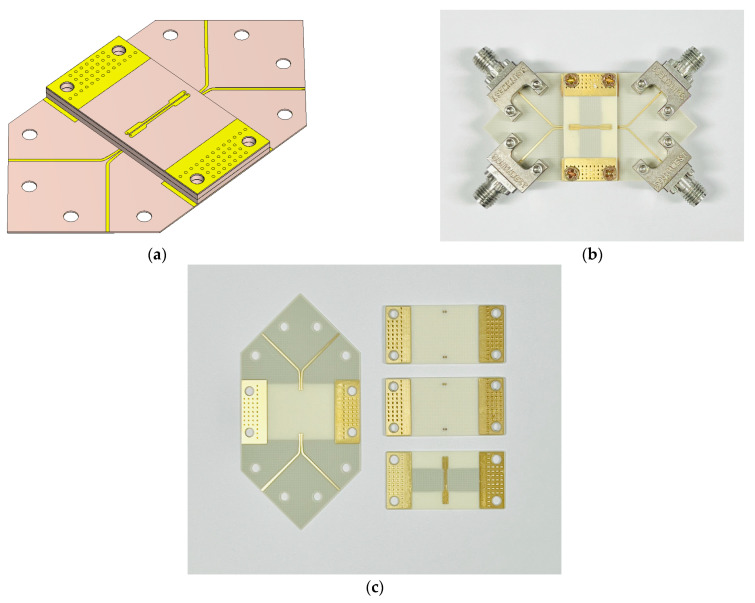
(**a**) Perspective view of the proposed CPS-based vertical transition modelled by the 3D EM simulator; (**b**) Fabricated CPS-based vertical transition in the back-to-back configuration; (**c**) Three circuit boards consisting of the proposed transition.

Figure 10a,b show the designed and fabricated CPS-based vertical transition, respectively. A back-to-back structure of the proposed transition is used to measure the properties of differential signal transmission using a 4-port vector network analyzer (VNA; Rohde & Schwarz ZNB40). To measure the 4-port S-parameters with the VNA, at the input and output ends, the gap distance between the two signal lines of the DL is gradually increased and transformed into two separate microstrip lines. The measured 4-port S-parameters are converted to mixed-mode S-parameters through de-embedding techniques. In this paper, the AITT (Advanced Interconnect Test Tool, Version 2022.08.17.) software is used to remove the effects of the measurement structures through de-embedding, which provides the 2X-Thru SFD method with automatic error correction [21].

Figure 10c shows the three circuit boards used in the fabrication of the transition. The CPS-based vertical transition is fabricated on two 32 mil thick dielectric substrates and one 8 mil thick dielectric substrate. The bottom substrate, fabricated on an 8 mil thick dielectric substrate, serves as a motherboard and provides four signal lines for the measurements with the 4-port VNA. The dielectric substrates are bonded together using silver epoxy paste, and to ensure stable bonding of the transition, they are secured with vertical connection screws. The overall size of the proposed vertical transition is 586 mil × 1100 mil × 85.6 mil.

**Figure 11:**
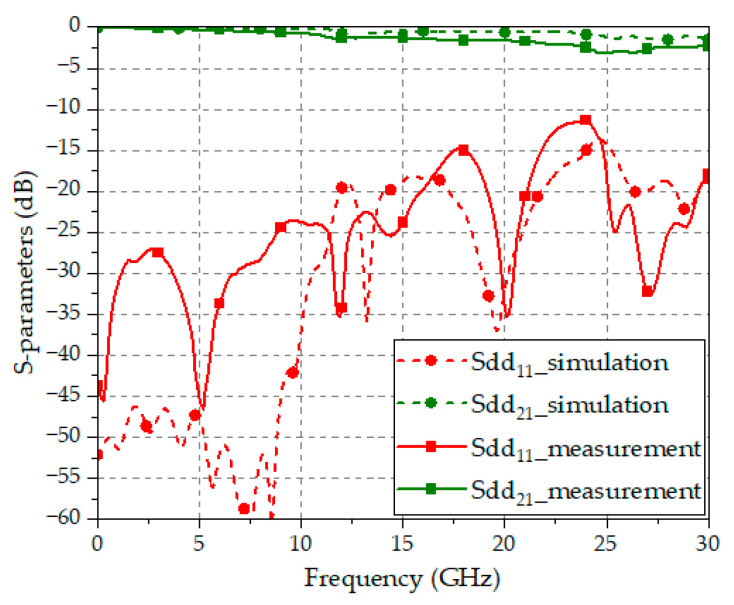
Measured and EM-simulated S-parameters of the proposed CPS-based vertical transition.

Figure 11 shows the measured S-parameters (${\mathrm{Sdd}}_{21}$ and ${\mathrm{Sdd}}_{11}$) of the proposed transition, compared with the EM-simulated S-parameters. These measured results include both the DL-to-CPS transition and the vertical via transition in the back-to-back configuration. The contribution of the DL-to-microstrip transitions for the 4-port measurements is removed through the de-embedding process. The measured results show a minimum return loss of 11.4 dB and a maximum insertion loss of 1.6 dB per transition in the frequency range of DC to 30 GHz. The EM-simulated results provide a minimum return loss of 13.9 dB and a maximum insertion loss of 0.8 dB per transition. The measured results exhibit a slightly increased insertion loss after 15 GHz as compared with the EM-simulated results. The slightly higher insertion loss with the measurements may have been caused by fabrication tolerances in the process of the manual bonding of the multiple dielectric substrates.

Therefore, the proposed CPS-based vertical transition successfully demonstrates its ultra-wide bandwidth exceeding 30 GHz, i.e., about three times the bandwidth of the conventional DL-based via structure. This enables more than 120 Gbps of digital data transmission with 4-level pulse amplitude modulation (PAM4) [22]. Furthermore, the proposed vertical transition is compatible with typical DL-based digital circuits, allowing its application in interlayer connections of high-density PCBs and interface junctions for ultra-high-speed digital data transmission.

## 5. Conclusions

In this paper, an ultra-wideband CPS-based vertical transition for ultra-high-speed digital interfaces is proposed. A differential line (DL) is typically employed in the transmission of high-speed digital data in commercial digital circuits. These DL-based digital circuits encounter issues like the effects of signal skew, FR4 fiber weave, and unbalanced EM interference due to the structural and electrical limitations of DLs. Particularly in the sections utilizing DL-based via structures for vertical line transitions, the complex structure makes it challenging to maintain constant line impedance and smooth electric field transformation, resulting in a limited frequency range (<10 GHz). On the other hand, a coplanar stripline (CPS), a type of planar balanced line (BL), can overcome many of these inherent issues of the DL, and secure an ultra-wideband frequency range with a relatively simple via structure, enabling ultra-high-speed digital data transmission.

The proposed CPS-based vertical transition comprises DL-to-CPS transitions and vertical via transitions. The design process of the transition is very efficient by using the analytical design formulas derived using the conformal mapping method. The characteristic line impedances of the transition, calculated using the analytical design formulas, closely match the results from EM simulations, and based on this, the CPS-based vertical transition maintaining the characteristic line impedance of 100 Ω is designed and fabricated. The measured results confirm its ultra-wideband characteristics, with a return loss of more than 10 dB from DC to 30 GHz. Therefore, the proposed CPS-based vertical transition is proven to offer a significantly wider frequency bandwidth, i.e., more than three times the bandwidth of the conventional DL-based via structures. This proposed transition may serve as a key technology for next-generation high-speed signal transmission, enabling significantly improved transmission speeds.

## Figures and Tables

**Table 1:** Comparison of the proposed vertical transition with the reported via structures.

Reference	Configuration	Frequency Range [GHz]	Number of Vias
[10]	DL-based vias	DC-16	4
[13]	DL-based vias	DC-20	8
[14]	DL-based vias	DC-7	4
This work	CPS-based vias	DC-30	2

**Table 2:** Line capacitance of each analysis region.

Region	Capacitance (*BB′*-*CC′*)	Capacitance (*FF′*-*GG′*)
I	$C_{1}{=\varepsilon }_{0}\varepsilon_{r}\frac{{K(k}_{1}^{\prime })}{{K(k}_{1})}$	$C_{1}^{\prime }{=\varepsilon }_{0}\frac{{K(k}_{1}^{\prime })}{{K(k}_{1})}$
II	$C_{2a}=\frac{\varepsilon_{0}\varepsilon_{r}}{2}\frac{{K(k}_{2a}^{\prime })}{{K(k}_{2a})}$ $C_{2b}{=\varepsilon }_{0}\varepsilon_{r}\frac{{K(k}_{2b}^{\prime })}{{K(k}_{2b})}$	
III	$C_{3}{=\varepsilon }_{0}\varepsilon_{r}\frac{{K(k}_{3}^{\prime })}{{K(k}_{3})}$	$C_{3}^{\prime }{=\varepsilon }_{0}\frac{{K(k}_{3}^{\prime })}{{K(k}_{3})}$
IV	$C_{4}{=\varepsilon }_{0}\frac{{K(k}_{4}^{\prime })}{{K(k}_{4})}$	$C_{4}^{\prime }{=\varepsilon }_{0}\varepsilon_{r}\frac{{K(k}_{4}^{\prime })}{{K(k}_{4})}$

**Table 3:** Design parameters of the proposed CPS-based vertical transition.

**Parameters**	*w_d_*	*g_d_*	*w_c_*_1_	*g_c_*_1_
**Size in mil**	11.4	5	13	5
**(mm)**	0.29	0.13	0.33	0.13
**Parameters**	*w_c_*_2_	*g_c_*_2_	*r*	*g*
**Size in mil**	24.5	5	6	29.1
**(mm)**	0.62	0.13	0.15	0.74

## Data Availability

The original contributions presented in the study are included in the article. Further inquiries can be directed to the corresponding author.
